# Anti-COVID-19 Vaccine Narratives Among African and North American Neo-Pentecostals (Part 2): Public Health Impacts and Theological Response to Leadership Gaps and Lessons

**DOI:** 10.1007/s10943-026-02685-4

**Published:** 2026-05-26

**Authors:** Daniel Orogun

**Affiliations:** 1 Department of Religion Studies, University of Pretoria, Pretoria, South Africa; 2 Centre for Studies in Religion & Society, University of Victoria, Victoria, BC Canada; 3 UC Davis Medical Centre, University of California, Sacramento, California USA

**Keywords:** Anti-vaccine narratives, Spiritual leaders, Theology of medicine, Public health, Africa and North America, Neo-Pentecostals

## Abstract

This study concludes a two-part interdisciplinary study examining the relationship between religious beliefs, actions, and health behaviors. Through a literature review, secondary data, and available media narratives, it examines the reactions of some African and North American Neo-Pentecostals during the pandemic. Part 1 revealed that the causes of anti-COVID-19 narratives, resistance, and hesitancy are historical, social, economic, theological, and technological (new media) (Orogun, 2026). This final exercise (Part 2) responds to the narratives and causes from public health impact and spiritual leadership perspectives. The public health implications discussed include (1) increased mortality and leadership vacuum, (2) families and communities’ grief, trauma, and emotional distress, and (3) broken trust in the government and health authorities. The spiritual leadership response articulated robust theological precedents in times of plagues and related health crises to show (a) the theology of medicine that shaped the worldview of leaders in biblical history and (b) the algorithm of strategies they deployed to manage plagues and related health challenges. Finally, lessons are presented to close the anti-COVID-19 narrative gaps in view of possible recurrence.

## Introduction

In part one of this study, literature reviews and statistics from academic and media spaces reveal evidence and causes of anti-COVID-19 narratives and hesitancy (Orogun, 2026, 1–19). The study further shows how the causes influenced the health behavior of Neo-Pentecostal leaders and their followers in Africa and North America. The historical, social, economic, theological, and technological (new media) causes further reveal poor strategic leadership in crises (Orogun, 2026, 20–21). The following paragraphs articulate eight lessons drawn from the narratives and causes discussed in Part One of the study.

First, anti-COVID-19 narratives and resistance existed long before the pandemic, and this foundation has increased current resistance to vaccines, which could also affect vaccination compliance in a future pandemic outbreak. Second, resistance to vaccination is not only influenced by Christian Religion and Neo-Pentecostal worldviews, but it also cuts across religions.

Third, religion is not the only reason behind vaccine hesitancy and resistance; moral, socio-cultural history, and existential issues like racism vis-à-vis population reduction contribute to influencing health behavior in a religious ecosystem. Fourth, while economic factors and the quest to consolidate spiritual authority may be the strongest motivations for the spiritual leaders’ theology of medicine and health behavior, public distrust of health stakeholders like Big Pharma and the Government on issues of race, population reduction, and threats to human freedom made it easy for the populace to choose their spiritual leaders over healthcare professionals and health authorities.

Fifth, the shortage of healthcare with rising demand and low supply influenced Neo-Pentecostal adherents to focus on spiritual alternatives to medicine. Sixth, poor theology of medicine and the influence of spiritual authority are behind the dominant and demonizing narratives against vaccines. Seventh, poor leadership and management skills during pandemics or related crises increase mortality and fatality rates.

Lastly, the research shows that consultations on the vaccine and related healthcare protocols between the government health authorities and the Neo-Pentecostal leadership are unpopular. Since the spiritual authority of the Neo-Pentecostal leaders influences health behavior, high-level consultation is essential as a counter-narrative to promote a positive response to vaccination (Orogun, 2026, 20–21).

With this background, this article aims to respond to some of the outcomes of the narratives and causes in two ways. First, the public health implications of the narratives and causes are presented. Second, the theology of medicine that shaped the worldview of leaders in biblical history is discussed. This is intended to be an ideal theology of medicine as a critique and possible response to prepare for future pandemics. Third, the theology of medicine will also show the algorithm of strategies deployed in biblical history to manage plagues and related health challenges. Finally, lessons are presented to close the anti-COVID-19 narrative gaps and help all stakeholders in the context to be well prepared to better manage emergency health crises or related pandemics in the foreseeable future.

The discussion in this research will also be incomplete without describing Neo-Pentecostals in context. Although Neo-Pentecostalism emerged in the 1950s and 1960s, the versions in context are contemporary Neo-Pentecostals from the 1980s to the present (Orogun & Pillay, 2022, 2). This group represents a modern charismatic reformation movement with a distinctive theology of spirit baptism that gives doctrinal priority to the gift of tongues (Orogun & Pillay, 2022, 3). While this paper has been made to be readable independently, it is also suggested that reading Part One will help readers grasp the line of thought in this paper. Now that the causes, lessons, and context have been provided above, the sections below will discuss the public health implications.

## Public Health Implications

The first part of this research discussed the narratives that cause anti-vaccine misinformation and pushback on compliance with health authorities. Thus, the first part covered the causes, while this section discusses the implications of these narratives for public health.

Drawing from the examples and scenarios from literature reviews and narratives in Part One of this research, some public health implications of vaccine hesitancy, pushback, and rebellion against health authorities’ protocols include increased mortality, panic, trauma, and hesitancy among congregants and communities. Likewise, distrust of the government, healthcare professionals, and scientific solutions to the pandemic increased following these narratives.

Furthermore, when pastors and members disobey healthcare protocols and vaccination mandates, the spread of the virus increases. Thus, pastors and congregants opened the door for increased infection in churches and communities and the eventual death of people in the community. This poses a substantial public health risk. Such a fast and easy spread of the virus is possible because some church leaders control millions of followers in Africa and North America. The public health implications are discussed in the following subsections.

### The Increased Mortality of Christian Leaders Created a Vacuum During and After the Pandemic

When Temitope Balogun Joshua prophesied that the pandemic would end after heavy rainfall in China, millions of his followers believed him and waited for the rainfall to wash away the virus rather than accept vaccination and related protocols (BBC, 2021). Such actions can put the lives of the communities and neighbors of TB Joshua followers at risk of illness and possibly death. In addition to prophecies, the stories detailed in part one of this study, under the section titled Causes of Misinformation and Claims: Narratives II, resulted not only in the deaths of congregants and communities but also in the significant loss of pastors’ lives (See Orogun, 2026, 8–20).

In Africa, some records include the death of Stephen Lungu, a Malawian African evangelist and former leader of African Enterprise (AE), who passed away after being hospitalized due to COVID-19 complications (Weber, 2021). In Durban, South Africa, Pastor Krish Govender tested positive for COVID-19 and died a few months after testing positive (Lutchman, 2021). While Pastor Frankline Ndifor of Kingship International Ministries in Cameroon prided himself on the spiritual ability to cure COVID-19, the clergy got sick and died from the virus. In Nairobi, Kenya, Reverend Mathew Wambua succumbed to COVID-19 complications and died after seven days of hospitalization (Wafula, 2021).

In Nigeria, Danjuma Tafa-Balewa, the pastor of the Redeemed Christian Church of God (RCCG), Cornerstone Parish in Okota, Lagos, died of COVID-19 complications after two weeks of hospitalization (Bolarinwa, 2023). In South Africa, three healthy young pastors, including Pastor Manyathela, passed away suddenly from COVID-19 health issues (Blessedman International, 2021). These few documented deaths of pastors in Africa may suggest a shortage of pastoral leadership and consequent wandering of sheep without shepherds during and after the pandemic.

In America, the founder of the Crenshaw Christian Center, Frederick K. C. Price died after COVID-19 complications (BBC, 2021; Althouse & McCormick, 2021). The Church of God in Christ (COGIC) in America only took the path of no gatherings after many deaths occurred among pastors and congregations across America (Boorstein, 2020; Crary, 2020). According to Pew Research, 1% of American adults identify as COGIC members (Pew Research Center, 2025). If youth and children are counted, the population of COGIC members could be approximately 6 million. Holding onto prayer as the primary and only response to COVID-19, as submitted by the General Overseer, implies that the church’s ‘theology of medicine’ could have influenced vaccine hesitancy and increased the mortality rate during COVID. In March 2020, COGIC held conferences in more than 300 locations, and members were encouraged to attend funerals held within the denomination. Bishop Porter believes that COVID-19 deaths may have spread from these events (Mattingly, 2020; Peagler, 2020).

In summary, the submission of this article shows that the gathering of churches and religious leaders’ decisions to sustain their authority and increase their revenues worked against the health authorities’ protocol and vaccination. The continuous gathering and spreading of false narratives increased the threat and spread of COVID-19 infections and resultant deaths in Africa and North America. This jeopardizes public health and safety.

### Families and Communities’ Grief, Trauma, and Emotional Distress

The death information provided above caused more family and community trauma and grief than the other causes. In South Africa, Pastor Govender’s wife and the church family in Durban were not only grieving over the clergy’s passing but also regretted being unable to provide a grand funeral ceremony; they last saw Govender on an admission bed before the sad event (Lutchman, 2021). Likewise, panic grips church members and the surrounding communities when Pastor Franklin dies of COVID-19 complications.

In Canada, family members grieved over the deaths of loved ones who defied health protocols and attended Pentecostal churches during the pandemic (Barry, 2021). The Church of God in Christ’s 6 million members nationwide also mourned the loss of several bishops and leaders in the United States due to COVID-19 complications (Mattingly, 2020; Peagler, 2020).

Pastor Gilyard also claimed the loss of four members to COVID-19, and twenty-six individuals were sick to the point of death. In Gilyard’s words, “There was so much death, disease, and hospitalizations that people of faith became weary and tired. They did not want church or even worship music because they were too depressed from the season” (Althouse & McCormick, 2024, 84). By implication, death and hospitalization during the pandemic threw many families and communities into trauma because depression and weariness are by-products of grief and trauma.

Besides grief and trauma within families and communities, global media news of death and the spread of the virus continued to create emotional distress and related mental health crises. Furthermore, different narratives on the danger of vaccines and rumors that labeled the pandemic ills as the anti-Christ agenda continued to create emotional distress across the globe. For example, Josh Hayden claimed that many spiritual leaders and followers were emotionally spent after intense discussions about race and the coronavirus (Goldberg, 2021). Hayden states, “They are really tired of addressing complicated issues and many are worn out” (Goldberg, 2021, 1). In other words, people were stressed, fed up, and losing their sanity because of the pandemic.

Therefore, this article submits that when grief and trauma take hold of communities and congregations because of the impact of the pandemic, public health and safety are jeopardized as mothers, children, fathers, friends, and neighbors are thrown into mourning because of the death of their loved ones. At the same time, they are scared for their lives.

### Broken Trust in Government and Health Authority

One significant implication of anti-COVID narratives was the distrust of the government and health authorities. Based on the secondary data in part one of this research, most congregants trust their spiritual leaders’ narratives on the pandemic. With such influence, the integrity of the government and health authorities regarding the science behind vaccination and related health protocols was severely damaged.

Since some of these leaders propagate anti-vaccine narratives to consolidate power and prosperity, their motives for anti-vaccine narratives are questionable. The spiritual leaders’ intentions were fulfilled, but public health and safety suffered a setback because there was a shift in trust from health authorities to spiritual authorities. The denigration of COVID-19 protocols encourages gatherings, laying of hands, trust in prophecies, and prayers against the virus without observing health safety. Thus, the church became one of the major spreading agents of the virus, leading to illnesses, deaths, grief, pain, fear, trauma, mourning, and mental health crises in various African and American communities.

Although there could be more implications that other researchers could investigate, the three subsections above reveal pieces of evidence of the public health implications of anti-COVID-19 narratives. The following section provides a theological response to the causes of the anti-vaccine narratives among African and American Neo-Pentecostals.

## Ideal Theology of Medicine as a Critique and Response to Probable Future Pandemics

In Part One of this study, one of the three main objectives highlighted was the ongoing debate about the potential for another outbreak in the coming years. According to some researchers, there is a 10–15% chance of another pandemic occurring within the next four years, and the world remains unprepared (Orogun, 2026, 2; cf. Dyos, 2025). Similarly, Bajeli-Datt’s report on Southeast Asia forecasted the return of the COVID pandemic in five years (Bajeli-Datt, 2025). Against this backdrop, this section aims to address the central question of how religious groups, particularly Neo-Pentecostals, should respond to a future pandemic if these predictions hold, while also critiquing the anti-COVID-19 narratives, actions and implications. To explore this question, this study presents theological narratives on how biblical leaders dealt with plagues and similar crises, using these as a lens to critique past leadership during the COVID-19 pandemic and to propose strategies for the future.

Looking at biblical history vis-à-vis the narratives presented in part one of this exercise, one clear distinction is that biblical leaders during plagues and diseases often relied on a combination of faith, prayer, leadership, and practical measures. This article considers practical measures, such as positive healthcare protocols and leadership strategies, to limit societal impacts. Contrarily, some religious leaders among the Neo-Pentecostals threw caution to the wind based on self-centered motives, thereby threatening public health and safety. The following subsections attempt to critique the Neo-Pentecostals’ leadership approach during the pandemic by citing scenarios of the ideal theology of medicine. The idea is to draw readers’ attention to the possibility of using these biblical scenarios as strategies to face possible future pandemic challenges.

### The Case of Leprosy and Furuncles as Examples of Poor Leadership in a Pandemic

In the story of King Uzziah, according to 2 Chronicles 26:16–21, the king was prideful and entered the temple to burn incense, a role reserved for priests. When criticized, Uzziah reacted with arrogance and anger toward his subjects. Consequently, the king was afflicted with leprosy. Rather than repenting, Uzziah remained adamant and isolated until his death. The king’s actions imply poor judgment of the dichotomy between the priests’ and the king’s roles.

An attempt to view the scenario above through the lens of clergy versus physicians’ responsibilities provides a precursor to why pastors died during COVID-19, while trying to fulfill the role of healthcare professionals by providing spiritual services like laying of hands and physical meetings during the pandemic. Switching responsibilities due to a sense of superiority and disregard for healthcare protocols shows that the poor judgment and decision-making of spiritual leaders added to the negative impact of the pandemic.

Similarly, during the plagues in Egypt, there was an outbreak of boils (furuncles). The situation came as a divine judgment when Pharaoh held the Israelites in captivity (see Exodus 9:8–11 and Deuteronomy 28:27). Flouting the instructions to release the Israelites, Pharaoh repeatedly hardened his heart despite the suffering of his people. His arrogance and inhumane approach to the well-being of Israelis and Egyptians suffering from the plague exacerbates the crisis.

Applying the Egyptian plague scenario to Neo-Pentecostal leaders’ approach during the pandemic, it can be inferred that many pastors acted as Pharaohs, hardened their hearts against vaccination, insisted on gathering, and jettisoned the healthcare protocols. Such actions, which prioritize leaders’ revenue and power-play (superiority over medical practitioners), suggest that the health and well-being of congregations and communities matter less, just as the plagues of Egypt matter less to Pharaoh. This reflects poor leadership judgment during health emergencies.

The two scenarios above are examples of poor leadership during pandemics. They reveal that poor leadership in healthcare is not novel to the field of theology. Since possible leadership failures have been briefly established, the following subsections articulate some theological examples of responsible spiritual leadership during health crises.

### Moses's Strategic Leadership for Plague Containment: A Type of Social Distancing and Mask Usage

Moses explored the spiritual and practical protocols during the plague. Moses prayed and repented for God’s mercy over the land and people (Numbers 16:46–50). In addition to prayers, Moses followed anti-plague protocols (instructions from God), which included marking doorposts with lamb’s blood during Passover (Exodus 12). Marking the doorpost with the lamb’s blood, as an instrument of ‘spiritual covering’ during the plague, can be likened to the mask used as an instrument of protection during the COVID-19 pandemic.

Moses also led the people to observe community separation as a quarantine measure. For example, lepers were isolated outside the camp to prevent the spread of leprosy as a contagious disease (Leviticus 13). This preventive measure can be likened to social distancing and isolation during pandemics. Moses’s strategic leadership during the plagues and contagious leprosy is a biblical standard for religious leaders’ responses to health crises, both in the past and future. Moses’s leadership evidenced that the government’s and health authorities’ approach was acceptable in the biblical strategy for disease containment. In this sense, spiritual leaders should not lead followers to rebel against health authorities, thereby creating public health and safety risks, but rather adopt Moses’s strategic leadership pattern in health crises.

### Joseph and David: People-Centered Leadership in Crises

Joseph prepared for the famine in Egypt by interpreting Pharaoh’s dream and implementing a strategic plan, storing surplus grain during years of abundance to sustain the population during the crisis. Joseph avoided mortality by hunger. His vision was to save the people from death (Genesis 41:48–49). Joseph made some sacrifices to prevent the hunger pandemic. The focus was on people’s well-being, not personal enrichment, and the abuse of position, as seen among some Neo-Pentecostal pastors during the COVID-19 pandemic. A case in point in the literature review in part one of this exercise was how a Neo-Pentecostal pastor in Canada got vaccinated to protect himself but never advocated for or spoke about the vaccine to his members (Orogun, 2026, 20–21). Ideally, religious leaders should sacrifice their desire to gather crowds and sustain their revenue generation by being more people-centered.

During the plague in Israel**,** David acknowledged his mistakes, repented, and sought God’s mercy. He made sacrifices. This could mean sacrificing his aggrandizement in the interest of members and communities. David took responsibility during the plague by building an altar to the Lord and offering sacrifices to stop the plague (2 Samuel 24:17–25). Again, David’s leadership style is people-centered. The approach to the pandemic was about the people. The management strategy here focuses on community well-being, not personal desires in crises.

### Noah’s Early Warning System and the Building of the Ark as Safety Protocols During the Flood Crisis: Evidence of the Connection Between Spiritual Action and Health Protocol

Noah’s survival strategy during the flood crisis in Genesis 6:9–9:17 reveals meticulous preparation based on obedience to God’s instructions (a spiritual action) to develop a safety strategy by building the ark and ensuring provisions for families and animals. Rabbi Danny Burkeman explains that Noah’s warning template was a strategic blueprint from God (Burkeman, 2019). Noah had a one-year ‘Early Warning Blueprint’, similar to the Early Warning, Alert, and Response Network (EWARN) in the medical field (World Health Organization, 2026). Based on the strategic blueprint, Noah provided ‘Early Warning Signs’, disseminated safety information, and provided the ark before the commencement of the flood (See Gen. 6:5; Moses 8:18–26).

In this article, the author sees the ark of safety as a quarantine station to separate people from the flood. In the COVID-19 pandemic context, it can be seen as (a) an isolation strategy and (b) personal protective equipment (PPE). Noteworthy is the depth of the interaction between God and Noah, a proof of Noah’s relationship with God and quality prayer life (Genesis 8:15, 9:17). Nevertheless, Noah did not depend solely on prayers during the flood crisis; he developed a strategic blueprint to save people from calamity. This classical scenario faults many Neo-Pentecostal leaders’ approaches to the COVID-19 pandemic, as delineated in part one of this research. For future purposes, Noah’s idea shows the need to deploy spiritual and medical strategies to address the coronavirus pandemic.

In another interpretation, Terris King, pastor of Liberty Grace Church of God in Baltimore, cited the story of Noah’s Ark to discuss common sense regarding pandemic decision-making. In Terris’s opinion, God utilized the science of the raven and the dove to determine when it was safe for Noah and his family to enter the Ark, which provided them with isolation and safety. Likewise, Terris used the story of Jesus distancing himself from his closest disciples before his death on the cross to illustrate a scriptural basis for social distancing (Sokolow, 2020). Thus, Noah’s safety mechanism during flood crises has a theological context for healthcare prevention and safety during pandemics.

### Ideal Theology of Medicine: A Hybridity of Spirituality and Medical Care in Health Crises

There is long-term tension between spiritual and medical leadership authorities. One of the reasons why Neo-Pentecostal leaders may have rebelled against vaccination and health care protocol is their philosophy of superiority over medical professionals and health authorities. The notion that religious individuals and communities should defer to spiritual authorities rather than medical practitioners is a misconception. It shows the level of subjugation and manipulation with which spiritual leaders rule the social and medical consciousness of people. In the ideal theology of medicine, the role of a physician is well-defined throughout biblical history, without any tension between the clergy and physicians. Therefore, there is no reasonable basis on which adherents should be influenced to uphold anti-vaccine ideas or to jettison health protocols during the pandemic.

The word ‘physician’ appears several times in the scriptures. In most Bible translations of the Old Testament, Genesis 50:2 mentions physicians who embalmed Jacob under Joseph’s directive. At the same time, in the New Testament, Luke is called ‘the beloved physician’ (Colossians 4:14). In 2 Chronicles 16:12, King Asa consulted physicians for his foot disease. Prophet Jeremiah cried—“Is there no Medicine in Gilead…Is there no physician there?” (Jeremiah 8:22—New Living Translation). Bible Hub commentary explains that **“In ancient times, physicians were expected to provide remedies for physical ailments, paralleling spiritual leaders' roles in addressing spiritual maladies”** (Bible Hub, 2025, 1). This statement, with particular emphasis on “paralleling,” suggests a side-by-side function of physicians and spiritual leaders.

Correspondingly, Perrine in Calvin Commentary states that **“Healer, or physician, is rendered ‘ἰατρὸς — healer,’ by the Septuagint, and ‘medicus’ by the Vulgate, Syriac, and Arabic. It appears that Gilead was not only celebrated for its healing gum but also for its medical men”** (Perrine, 2009, 1). These words affirm the recognition of medical practitioners’ role in the Bible; a practice that should subsist even now.

Furthermore, Jeremiah 8:22 is metaphorical because it speaks to people’s spiritual and physical ailments. Across Bible translations, the ‘daughter of my people’ in the text refers to the people of Judah in suffering and their need for restoration. Jeremiah was busy questioning why they had not leveraged the healing available to them physically (medical) and spiritually (Bible Art, 2025).

The most important New Testament example is Jesus’ understanding of the role of physicians. Colossians 4:14 states that Luke was a medical practitioner. Although Luke worked with Jesus, his profession could not have been completely irrelevant to the Gospel. As the case may have been, Luke could have served spiritually and medically. Matthew 9:12 also suggests that Jesus implied that the sick need a doctor, not only a pastor’s instruction.

Overall, the biblical recognition of physicians should send a message to Neo-Pentecostal leaders to recognize and support the role of medical practitioners and allow their followers to leverage vaccination and related health protocols during the pandemic to encourage public health and safety.

### Jesus' Scientific Approach to Spiritual Healing: A Typology of Testing in the COVID-19 Treatment Protocol

The New Testament provides a good example of human-centered leadership during a health crisis, where Jesus performed evidence-based healing. In Luke 17:11–19, Jesus had ten patients suffering from leprosy, a contagious disease requiring isolation, just like COVID-19. In demonstrating compassion, courage, and spiritual authority as part of a healing practice, Jesus approached them, spoke words of healing, and instructed them to show themselves to priests to confirm their healing. This exercise could imply that Jesus subjected healing performance to review as a form of ‘testing’ to ensure that the results show a clean bill of health. The exercise could have been conducted to ensure a clean bill of health before the infected people mixed with the crowd or communities. Therefore, Jesus’ scientific leadership approach highlights empathy, proactive care, respect for health protocols such as testing, and embodies exemplary leadership during a health crisis.

Critiquing Neo-Pentecostal leaders’ approach through the lens of Jesus’ scientific testing protocol, it was clear that the refusal to test for COVID-19 infection by many adherents who came in contact with clergy who died of COVID-19 through laying of hands and physical gathering has no biblical precedent. A situation where followers of late pastors decline testing, as discussed earlier in the narratives, indicates a poor theology of medicine and crisis management strategy.

In the literature reviews provided in part one of this research, there are rarely any cases where those in contact with dead members and pastors because of gatherings or laying of hands are mandated to take COVID-19 tests before re-integrating with families, congregations, and communities. This shows a poor testing culture among Neo-Pentecostals and gives room for non-verified claims. Without such verification, the likelihood of spreading uncured diseases is very high, increasing the risk of infection and endangering public health and safety.

Overall, the implication is that Jesus Christ can be linked to scientific protocols in healing practices. Some Pentecostal leaders’ lack of understanding of the role of scientific protocols in health emergencies led to the demonization of anything scientific. For example, Allen Jack Coe taught his followers that **“The day would come when those who consulted physicians would have to take the ‘mark of the beast’ and that men were clearly looking to the wrong source for healing when they consulted doctors”** (Harrell, 1979, 101).

Another claim was made by Gloria Copeland, who invented the slogan ‘Word Inoculation’. This means that the word of God is the vaccine. Gloria denigrated flu vaccination with these words: **“We do not have a flu season and do not receive it when somebody threatens you with ‘everybody is getting the flu’”** (LaMotte, 2018, 1). For Gloria, Jesus was protected from the flu; therefore, Gloria’s listeners should avoid the virus by repeating the phrase, **“I’ll never have the flu”** (LaMotte, 2018, 1). This is a dangerous narrative antithetical to Jesus’s scientific approach to health crises, and it can increase the resistance of religious adherents’ subscription to vaccination.

## Limitations of the Study

This exercise (Parts one and two) does not claim that only Neo-Pentecostals disobeyed the healthcare protocols and died during the pandemic; leaders across denominations and religions also died**.** For example, a Milwaukee-born Catholic priest, one of the Vatican’s paramount experts on Latin, died on Christmas Day in a nursing home after testing positive for COVID-19. Another 49-year-old priest in Brooklyn was the first Catholic cleric to die of coronavirus in the United States. Rabbi Yaakov Perlow, 89, president of Agudath Israel of America, died in April due to complications arising from COVID-19 (Boorstein, 2020; Crary, 2020).

Rev. Franco Minardi, aged 94, was one of the scores of Italian priests who died of COVID-19. Patriarch Irinej, aged 90, was the Serbian Orthodox Church’s top leader. Irinej died after testing positive for the coronavirus. Rev. Dr. Ron Hampton of the Free Methodist Church in Shreveport, Louisiana, died due to COVID-19 complications (Boorstein, 2020; Crary, 2020). Consequently, other denominational leaders’ approaches to the pandemic can be explored by future researchers.

Although this study provided some secondary data in part one, it is more literary and historical in nature. Thus, there is an opportunity for other researchers to adopt a more evidence-based approach, with primary data collection and analysis, to prove how anti-COVID-19 narratives among religious leaders impact public health and safety.

Finally, the adverse effects of vaccines were not discussed in this study. This does not deny the possibility of side effects. Instead, it shows the limitations of the scope of the study and allows future researchers to investigate the role of spiritual leaders in addressing the side effects of vaccines.

## Conclusion

The narratives in part one of this exercise support the existence of anti-vaccines, poor leadership strategy in health crises, and public health implications among Neo-Pentecostals in Africa and North America during the pandemic. The narratives revealed the causes of anti-vaccine misinformation and pushback on compliance with health authorities. The causes included poor theology of medicine, social media and religious networks’ influence, inadequate leadership strategies in crisis management, the influence of religious leaders on adherents’ health behavior, the gap between healthcare demand and supply, the superiority tension between spiritual and healthcare leadership, and the consolidation of pastoral authority and prosperity.

Part two, presented in this article, further links the narratives to public health implications such as grief, trauma, and death in communities. Most importantly, this study responded to the call by Terris King, pastor of Liberty Grace Church of God in Baltimore, to fill the gap created by a lack of narration and review of health and spirituality stories to address the tension between health professionals, government, and spiritual leadership in faith communities. It provided some theological crisis management strategies and an ideal theology of medicine, and ended with lessons on how to prevent the potential spread and subsequent calamities in the future. The lessons for the future are presented below:

## Future Lessons

**(i) The role of healthcare professionals is recognized throughout biblical history:** There is no theological common sense in disobeying healthcare protocols, such as testing, social distancing, using masks, and vaccination, during a pandemic. The lesson here is the need for spiritual leaders to synergize with healthcare professionals in tackling future pandemics.

**(ii) The anti-vaccination narrative of Neo-Pentecostals in Africa and North America cannot be generalized:** Not every religious leader is against the COVID-19 vaccine and healthcare protocol. Some leaders in Africa and North America support the public health protocols available to avoid spreading the virus. Some preached vaccine acceptance as part of God’s healing measures (see Umc.edu, 2021).

**(iii) Anti-vaccine narratives and poor leadership approaches are not novel to theology but are unbiblical:** There are cases of poor leadership approaches to health crises in the Bible. Hesitancy and pushback were not in line with biblical leaders’ approach to plagues and related pandemics. This is a sign that many Neo-Pentecostal leaders misinforming the public were not predicated on theological accuracy. For future purposes, spiritual leaders must weigh their actions and decisions through the lens of the ideal theology of medicine.

**(iv) Anti-vaccination narrative is not exclusive to Neo-Pentecostals in Africa and North America:** Other denominations are not excluded from the narratives, including those who worked against vaccination based on politics and race (See Vann et al., 2021).

**(v) Non-Christian religious groups also spread misinformation about vaccination:** Unscientific narratives about COVID-19 and vaccination misinformation promoted by religious leaders appeared to spare no religious belief. In other words, other religious groups may be guilty of the antivaccine narratives (See Galang, 2021, 513–514).

**(vi) Delayed response leads to delayed safety and risk of public health misfortune:** Based on the narratives provided, there appears to be a change in the impact of COVID from the year 2022 onward. However, many lives would have been saved if these measures had been implemented earlier. This speaks to some spiritual leaders’ culture of response to health crises (See Belz, 2023; Moore et al., 2022, 8926). The lesson for the future is the need for faith communities to respond early to healthcare protocols.

**(vii) The anti-vaccination influence over congregants was not by religious leaders and groups alone, but also by the media, political views, and related conspiracies.** Some pastors who took the vaccine also found it difficult to convince their members to take the vaccine due to political and media influences that remain outside the control of church leadership (See Goldberg, 2021).

**(viii) Exemplary leadership, unified positive thoughts, and affirmation in health crises are imperative:** Some pastors agreed with health protocols and led by example as they took the vaccines publicly. The submission is that when pastors lead by example in taking vaccines, church adherents and related communities will easily respond to vaccination calls (Reeves, 2021). There are also differing opinions on vaccination during the pandemic. Unified positive thoughts among Neo-Pentecostal leaders on vaccination could help in future occurrences (Reeves, 2021).

**(ix) A ‘Testing Culture’ to verify healing needs to be developed in the faith community:** Jesus exemplified the need to test and verify health status after a spiritual exercise. Although spiritual healing may be supernatural, the need to verify the healing condition builds trust with the healer and safeguards the community from the spread of diseases.

**(x) The need to renew theological thinking in the post-COVID-19 era is imperative:** Renew is used intentionally because the idea of vaccination and compliance with health protocols predate the COVID-19 pandemic. Over the years, the values of hybrid healthcare between the spiritual and medical have lost relevance because of the rise of miracle healing evangelists among Neo-Pentecostals. In the future, revisiting and renewing the medical theology of Neo-Pentecostals in line with the medical theology discussed in the previous section may be helpful. This includes taking cues from the positive responses of the past.

For example, Billy Graham’s family acted positively during the 1918 flu pandemic. In the words of his son Franklin Graham, **“I think if there were vaccines available in the time of Christ, Jesus would have made reference to them and used them”** (Vann et al., 2021, 1). Robert Jeffress also called the vaccines a **‘**gift from God’ while hosting a vaccination clinic at his 14,000-member megachurch, First Baptist in Dallas. (Goldberg, 2021). Against anti-vaccination theology, Curtis Chang asked – **“Why not tell people that the Lord has already answered our prayers by giving us brains, and by giving us science?”** (Poitras, 2021, 1; cf. Guzman, 2021).

Likewise, Springfield Mayor Ken McClure said the region experienced a big jump in vaccinations after a pastor preached, and after more than 200 pastors, priests, and other church leaders from Missouri adopted the ‘love your neighbor as yourself’ principle to mobilize members and communities to accept vaccination (Reeves, 2021).

These are great examples of renewed theological thinking during health emergencies. The lesson is to be flexible, put on a thinking cap, and renew theological responses to health emergencies.
